# Establishment and Characterization of Brain Cancer Primary Cell Cultures From Patients to Enable Phenotypic Screening for New Drugs

**DOI:** 10.3389/fphar.2022.778193

**Published:** 2022-03-17

**Authors:** Michelle dePadua, Preethi Kulothungan, Rahul Lath, Ravikanti Prasad, Kranthi Madamchetty, Shravya Atmakuri, Sravanthi Ragamouni, Mukesh Gandhari, Lakshmipathi Khandrika, Jugnu Jain

**Affiliations:** ^1^ Department of Pathology, Apollo Hospital, Apollo Health City, Hyderabad, India; ^2^ Saarum Sciences Pvt Ltd., Hyderabad, India; ^3^ Department of Neurosciences, Apollo Hospital, Apollo Health City, Hyderabad, India; ^4^ Department of Radiology, Apollo Hospital, Apollo Health City, Hyderabad, India; ^5^ Bioserve Biotechnologies India Pvt. Ltd., Hyderabad, India; ^6^ Mapmygenome, Madhapur, India; ^7^ Sapien Biosciences Pvt Ltd., Hyderabad, India

**Keywords:** glioma, desmoplastic infantile ganglioglioma, neurosphere, cancer stem cell (CSC), brain cancer cell, CD133+

## Abstract

**Aim:** Desmoplastic infantile ganglioglioma (DIG), is a rare tumor arising mainly during the first 2 years of life. Molecular characterization of these benign yet rapidly proliferating tumors has been limited to evaluating a few mutations in few genes. Our aim was to establish a live cell culture to enable the understanding of the cellular processes driving the non-malignant growth of these tumors.

**Methods:** Tumor tissue from a rare non-infantile 8-year-old female DIG patient was dissociated and digested using collagenase to establish live cultures. Both 2D monolayer and 3D neurospheres were successfully cultured and characterized for proliferative potential, intrinsic plasticity, presence of cancer stem cells and the expression of stem cell markers. Cells cultured as 3D were embedded as tissue blocks. Immunohistochemistry was performed in both tissue and 3D sections for markers including synaptophysin, vimentin, neurofilament and MIB-1. Mutation analysis by NGS was performed using a-100 gene panel.

**Results:** Using immunohistochemistry, the 3D cultures were shown to express markers as in the original DIG tumor tissue indicating that the spheroid cultures were able to maintain the heterogeneity found in the original tumor. Cells continued proliferating past passage 10 indicative of immortalization. Enrichment of cancer stem cells was observed in neurospheres by FACS using CD133 antibody and RT-PCR. Mutation analysis indicated the presence of germline mutations in three genes and somatic mutations in two other genes.

**Conclusion:** A spontaneous cell line-like cell culture with high percentage of stem cells has been established from a DIG tumor for the first time.

## Introduction

Desmoplastic infantile ganglioglioma (DIG), is a rare tumor arising mainly during the first 2 years of life. Non-infantile cases are rarer, and when reported can range from 5 to 25 years (Khaddage et al., 2004; Gelabert-Gonzalez et al., 2011; Chandrashekhar et al., 2012). These tumors were initially classified as composite cerebral neuroblastoma and astrocytoma and are now classified by WHO as neuronal and mixed glio-neuronal tumors (Bhardwaj et al., 2006). DIG is a grade I tumor involving superficial cerebral cortex and leptomeninges, often attached to dura, with a large mass composed of both solid and cystic areas. The tumor is heterogeneous with meningeal tumor cells, mainly a mixture of fibroblast like cells, and neuroepithelial cells in a background of connective tissue. Although these tumors are considered to be non-malignant, they have a high rate of proliferation and can reach large sizes before being diagnosed. In rare instances, DIGs have been reported to become malignant mostly by acquiring genetic mutations (Loh et al., 2011; Prakash et al., 2014).

The diagnosis is done using both radiological and histopathological analysis. Both DIG and DIA (Desmoplastic infantile astrocytoma) present as large mixed masses under contrast, predominantly containing cystic mass with peripheral solid structure. The two types are differentiated histologically where DIG has a mixture of both glial and neuronal markers while DIA has mainly glial markers upon histochemical analysis (Taratuto et al., 1982; Craver et al., 1999; Derinkuyu et al., 2015).

The radiological, clinical -and histo-pathological findings of DIG have been well described in literature (Paulus et al., 1992; Bader et al., 2015), but molecular and cellular characterization has been limited. Genetic alteration in BRAF (V600E mutation and V600D), has been reported in multiple cases (Gessi and Pietsch, 2013; Koelsche et al., 2014; Wang et al., 2018). In cases where multiple other mutations, in genes such as TP53, ATRX, and others, recurrence of the tumor is a possibility. Malignant transformation of DIG into GBM was observed in a case with a wild-type BRAF but with several other genetic abnormalities including large deletions in 4q and Y, amplification of 12q14 and a SNP (R248Q) in TP53 (Prakash et al., 2014). Other genomic alterations in chromosomal regions such as 4q12, 5q13.3 and 21q22.11 have been observed in some DIGs and the closely related DIAs where neoplastic astrocytes are observed instead of neurons in the case of DIGs (Gessi and Pietsch, 2013). These studies emphasize the importance of genetic analysis of these tumors for optimal treatment and follow-up.

In many of the cases, complete surgical resection is enough to completely cure the disease; however, where this is not possible, chemotherapy is suggested (Smith, 2006). In many cases, these tumors can reach large sizes and can lead to severe morbidity to the child due to the surgery. Alternate therapies that can reduce the tumor size *in situ* or total elimination of the tumor either as neo-adjuvant or adjuvant therapies are hence in demand.

The aim of this study was to obtain cultures of cells from the DIG tissue sample and characterize the cells for the presence of the same markers that were used for pathological diagnosis of the disease. In addition, the rate of proliferation of the cells, the intrinsic plasticity, lifespan, and presence of stem cells like features was also determined. It is our belief that these cells are a good model system for understanding the disease and its probable transformation into a malignant disease in addition to their usefulness in drug discovery and development. To our knowledge, this is the first successful attempt at culturing the cells from a DIG tumor tissue and the derivation of a spontaneous cell line-like proliferative cell culture.

## Methods

### Materials

All the chemicals and labware used were tissue culture grade and were procured from Gibco (Invitrogen, Carlsbad, CA) unless specified otherwise.

### Pathology

Tumor tissue received from the surgery was fixed in 10% neutral buffered formalin and processed. Immunohistochemical studies were done on Ventana benchmark XT (Adriamed Ltd., Skopje, North Macedonia). The antibodies used were GFAP (Clone: GA-5, ready to use, Biogenex Laboratories, Fremont, CA), Synaptophysin (Clone: GR007, ready to use; PathnSitu, Pleasanton, CA), Vimentin (Clone: V9, ready to use, DakoCytomation, Glostrup, Denmark), Neurofilament protein (Clone: 2F11, ready to use, DakoCytomation) and Ki 67 (Clone: MIB-1, ready to use. DakoCytomation). The mean Ki 67 index was calculated from 10 high powered fields, by counting the number of tumor cells with nuclear positivity per 100 tumor cells.

### Glioma Cell Culture

A small part of the surgically excised fresh glioma tissue was collected in sterile saline. Tumor sample was dissociated within 30 min of excision, using Collagenase Type IV (Sigma-Aldrich, St. Louis, MO) for 30 min. The reaction was stopped by diluting with cell culture medium containing 10% FBS and then strained through a 40 μ filter. The resulting cells were placed in a T-75 flask (2 × 10^6^ per T75 flask). Cells were initially cultured and maintained in DMEM/F12 medium supplemented with 10% fetal bovine serum, Penicillin-Streptomycin, Glutamax and incubated in 37°C in a humidified incubator with 5% CO_2_.

Normal human mammary fibroblasts were also cultured from breast-reduction surgeries. Fibroblast cells were isolated from normal breast tissue using 100 U/ml Collagenase type IV and 2 U/μl DNase after overnight digestion at 37°C in a 5% CO_2_ incubator. These cells were used as non-neuronal controls for cell staining.

For assessing senescence, monolayer cultures were washed with PBS and dissociated using 0.05% Trypsin-EDTA for 5–7 min. Approximately 5,000 cells were plated onto coverslips in a 6-well plate and incubated overnight at 37°C/5%CO_2_, washed with PBS and fixed using 4% paraformaldehyde. Cells were then incubated 12–16 h at 37°C with freshly prepared X-gal (1 mg/ml, 5-bromo-4-chloro-3-indoly *β*-D-glucose), 5 mM K_3_Fe(CN)_6_, 5 mM K_4_Fe(CN)_6_, 2 mM MgCl_2_ in citrate-buffer saline pH 6.0. At the end of the incubation cells were washed with PBS and examined at ×100 objective magnification using a light microscope (CK×41, Olympus Corporation, Tokyo, Japan).

### Spheroid Culture

For spheroid culture, cells grown in monolayer were trypsinized and counted by hemocytometer. Cultures were established using protocol described by Lee et al. (Lee et al., 2006), with slight modifications. Briefly, 0.5 × 10^6^ cells were plated on 60 mm non-adherent tissue culture plates (Eppendorf AG, Hamburg, Germany) with DMEM/F12 media supplemented with human EGF, human bFGF, N2 and B-27 supplement.

Neurospheres were collected and centrifuged at 500 rpm for 5–7 min and washed thrice with PBS. Accutase (Sigma-Aldrich) was added and cells triturated using a Pasteur pipette gently until the neurospheres were dispersed into single cells. The cell suspension was further incubated with the Accutase at 37°C in a shaking water bath for 5–10 min. Cells were washed twice with PBS and counted using hemocytometer.

### Flow Cytometry Analysis

Cells grown in 2D and 3D cultures were trypsinized, or subcultured using Trypsin and Accutase respectively as described previously. Cells were washed twice with PBS and distributed into 2 different tubes at 0.5–1 × 10^5^ cells per tube. Cells were either left unstained or stained with 2 μL of CD133-PE (Miltenyi Biotech GmbH, Bergisch Gladbach, Germany) antibody at 4°C for 20 min. After washing with PBS, cells were analyzed by flow cytometry for CD133 positivity (Li, 2013).

Viability of cells in spheroid culture were determined using fluorescent dyes (Calcein-AM and prodidium iodide) (Sutherland, 1988). Spheroid cultures at passage number 4, were collected into a conical tube, centrifuged at 800 rpm, washed with PBS and transferred gently to 8-well chamber slides. The spheres were stained with 0.1–1 μM Calcein-AM for determining live cells and Propidium iodide for dead cells. Cells were incubated for 30 min at 37°C in presence of the dyes and then analyzed by fluorescence microscopy (Eclipse Ti, Nikon Instruments, Tokyo, Japan).

Neurospheres of 100–200 μm size were embedded in egg albumin, placed in formalin and processed as FFPE blocks. Sections of 2 μm were cut and stained with Hematoxylin and Eosin according to standard protocols to observe the spheroid morphology. The sections were also stained for different markers by IHC and the staining intensity and distribution was compared with the DIG tissue FFPE sections.

### RNA Isolation and RT-PCR

DIG cells were cultured as adherent 2D and suspension 3D cultures for 7–10 days and RNA was isolated using Trizol reagent according to manufacturer’s protocol (Invitrogen). RNA (1 μg) was converted into cDNA using high-capacity cDNA reverse transcription kit (Applied Biosystems, Waltham, MA). PCR was carried out using the Mastercycler nexus (Eppendorf, Hamburg, Germany), according to the manufacturer’s instructions. The cycle conditions for PCR were 95°C for 2 min, followed by 30 cycles of 95°C for 15 s, 60–70°C for 30 s and 72°C for 1 min. The sequence of primers used is shown in Table 1.

**TABLE 1:** Primer sets used for PCR.

Gene	Forward primer (5′-3′)	Reverse primer (5′-3′)
hNANOG	AGT​CCC​AAA​GGC​AAA​CAA​CCC​ACT​TC	TGC​TGG​AGG​CTG​AGG​TAT​TTC​TGT​CTC
hOct4	GAC​AGG​GGG​AGG​GGA​GGA​GCT​AGG	CTT​CCC​TCC​AAC​CAG​TTG​CCC​CAA​AC
hSOX2	GGG​AAA​TGG​GAG​GGG​TGC​AAA​AGA​GG	TTG​CGT​GAG​TGT​GGA​TGG​GAT​TGG​TG
hKLF4	CCC​ACA​CAG​GTG​AGA​AAC​CT AC	ATG​TGT​AAG​GCG​AGG​TGG​TC

### Mutation Analysis

Sections of 5 micrometer thickness were used for extracting genomic DNA using QIAamp DNA FFPE Tissue Kit (Qiagen, Hilden, Germany). Mutation analysis was performed using the Bioserve NGS panel for SNP analysis in 100 genes.

## Results

### Clinical Characteristics and Diagnosis of DIG Tumor

**FIGURE 1:**
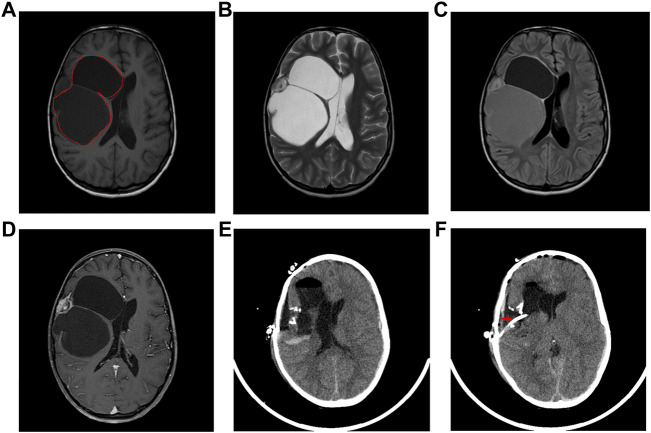
Radiological features of the tumor. **(A)** The tumor region is demarcated using red lines in an Axial T-1 weighted image, **(B)** T-2 weighted, **(C)** Flair and **(D)** T-1 contrast MRI showing a multiloculated tumor with solid and cystic areas causing mass effect on the ventricle with midline shift. **(E)** Post-operative axial CT scan showing tumor excision and decreased mass effect and return of midline structures. **(F)** Axial CT scan showing the catheter (marked by a red arrow) in the cystic cavity.

The DIG patient was an 8-year-old girl that presented with multiple episodes of jerky movements of all four limbs since the age of 5 years. There was a history of brief loss of consciousness following the last episode. She had developed weakness of upper and lower limbs since the last 2 years. Her higher mental function and cranial nerve examination was normal. She had left hemiparesis (4/5). Her sensory functions were normal. No signs suggesting cerebellar or meningeal involvement were seen. MRI of the brain (Figures 1A–D) showed a mixed intensity mass lesion in the right fronto-parietal region with mass effect on the ipsilateral ventricle and midline shift to the left. There were solid and cystic areas with calcification and enhancement of the solid component. A thinning of overlying frontal lobe was seen.

The patient underwent right fronto-temporal parietal craniotomy with excision of the tumor. Intraoperative findings revealed a SOL with the solid and cystic part pushing Sylvian fissure inferiorly. The frozen section of the tumor was reported as a low-grade glioma with desmoplasia. The post-operative CT scan had shown gross total excision of tumor with hemorrhagic fluid which was cleared in subsequent scans (Figures 1E,F).

**FIGURE 2:**
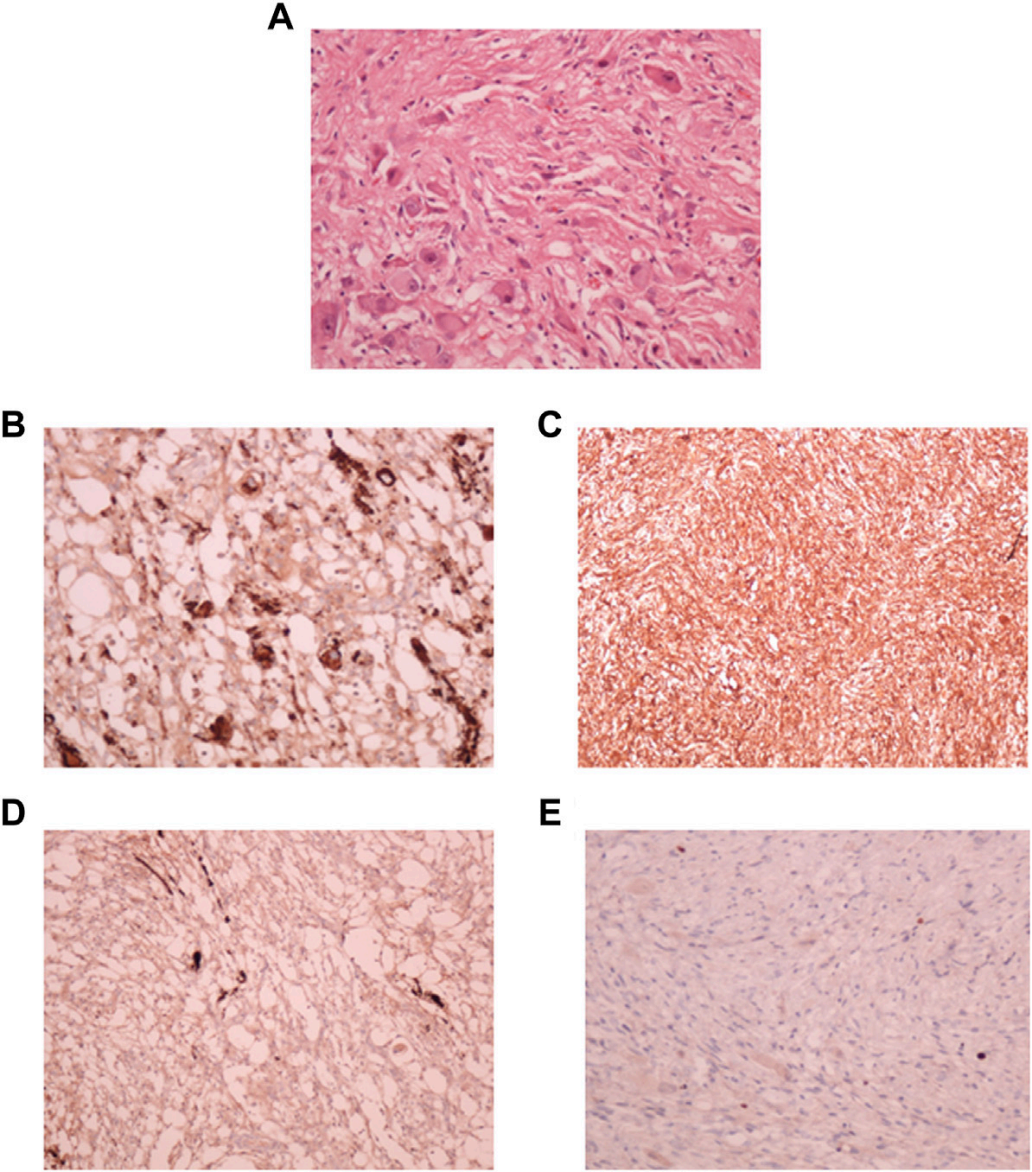
Pathological diagnosis of DIG. **(A)**: Hematoxylin and Eosin stained tissue section at 40X showing desmoplastic stroma with intervening atypical neuronal cells with enlarged nucleoli. Immuno-histochemical staining showed strong positivity for synaptophysin **(B)**, Vimentin **(C)**, focal positivity for GFAP **(D)**, and a lack of staining for MIB-1 index **(E)**.

Microscopic evaluation of the neoplastic tissue showed that it was composed predominantly of spindle fibroblast-like cells. Intervening atypical neuronal cells were seen, some with enlarged nuclei, prominent nucleoli, and abundant cytoplasm (Figure 2A). Scattered neoplastic astrocytes were identified with a few gemistocytic astrocytes. Necrosis, mitotic figures or endothelial proliferation was not seen. There was no evidence of poorly differentiated neuroepithelial cells.

Focal strong positivity for synaptophysin (Figure 2B), and strong diffuse positivity for Vimentin protein (Figure 2C) was noted upon immunohistochemical staining. Focal positivity for Glial Fibrillary Acidic Protein (GFAP) was also seen (Figure 2D). MIB-1 index was low, <0.5% (Figure 2E). Due to the observation of both glial and neuronal features by histological observation and marker expression, this tumor was diagnosed as a DIG.

### Establishment of DIG Cell Culture

**FIGURE 3:**
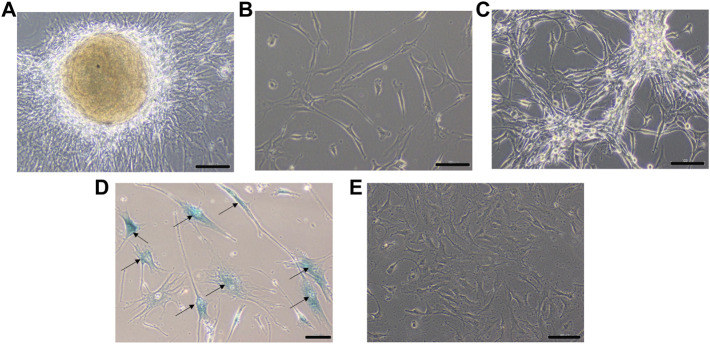
Phase contrast picture of DIG cell culture at different days of growth. Scale bars represent 100 μm. Morphologically, the cultures comprise a mixed population of cells including neuronal, glial and astrocyte cells. **(A)**: Tissue outgrowth of cells from a small piece of tissue adherent to the tissue culture vessel. **(B)**: Diffuse cell growth at day 6. **(C)**: Dense growth of cells at day 10. **(D)**: Human fibroblast cells at passage 5 showing intense blue stain with beta-galactosidase indicating senescence. **(E)**: DIG cells in culture at passage 10 showing a lack of senescence.

A small part of the fresh tumor tissue was used to establish an adherent cell culture. Cells were observed to be adherent and 40–50% confluent after 6 days. Morphologically they were identified as a mixed population of both neuronal and glial cells (Figure 3A). Though the initial density was lower at day 6 (Figure 3B), dense growth was observed by day 10 (Figure 3C), suggesting a fast rate of multiplication of the cells in adherent culture. The growth rate between passages 1 and 2; cells in passage 1 had a doubling time of 1.88 days while the passage 2 cells had 3.11 days. The isolated cells grew well in culture.

From the initial monolayer adherent cells, 3D suspension cultures were derived in serum-free special medium in low attachment plates at passage 2. These conditions have been known to favor the formation of multi-cellular suspension cultures or neurospheres. Formation of spheres was observed in 3–4 days with cell clusters continuing to increase in size over days with only a few single or dead cells. The rate of doubling of cells in 2D and 3D cultures was 2 and 7 days respectively indicating that either the cells doubled faster in serum-containing medium or that many cells were not able to form spheres and survive serum-free conditions in 3D cultures. The size and number of spheres in the culture remained same at least up to P#5, but subculturing spheroid cultures from 3D to 3D took longer and the cell yield decreased over serial passages.

Cells were passaged serially as monolayers up to 10 passages without any loss of proliferation. In our experience, primary cells typically have a finite life span *in vitro* and thus slow down and start becoming senescent by passages 5–6. In order to check for senescent cells, a beta-galactosidase assay was performed. Less than 5% cells were observed to be senescent at the 10th passage suggesting the establishment of a spontaneous DIG cell line (Figures 3D,E). In contrast, normal human mammary fibroblasts, cultured from elective breast-reduction surgeries at passage 5 were positive for the beta-galactosidase stain (Figure 3D) in the cytoplasm indicating their being senescent. More than 50% of the fibroblast cells showed senescence at the 5^th^ passage. Unlike DIG cells, these fibroblast cells do not proliferate further and eventually die in culture.

### Cells Cultured as Neurospheres Retain Expression of Tissue Neuronal and Glial Markers

In order to confirm that the cells in culture retained the characteristics of the cells in the tumor tissue, the expression of specific markers was assessed by IHC. The larger neurospheres (100–200 μm in size) were encapsulated in ovalbumin and fixed in formalin and embedded in paraffin like FFPE blocks. Sections obtained from these FFPE blocks containing the neurospheres were stained with H&E, GFAP, Synaptophysin, Neurofilament protein, MIB-1, and Vimentin.

**FIGURE 4:**
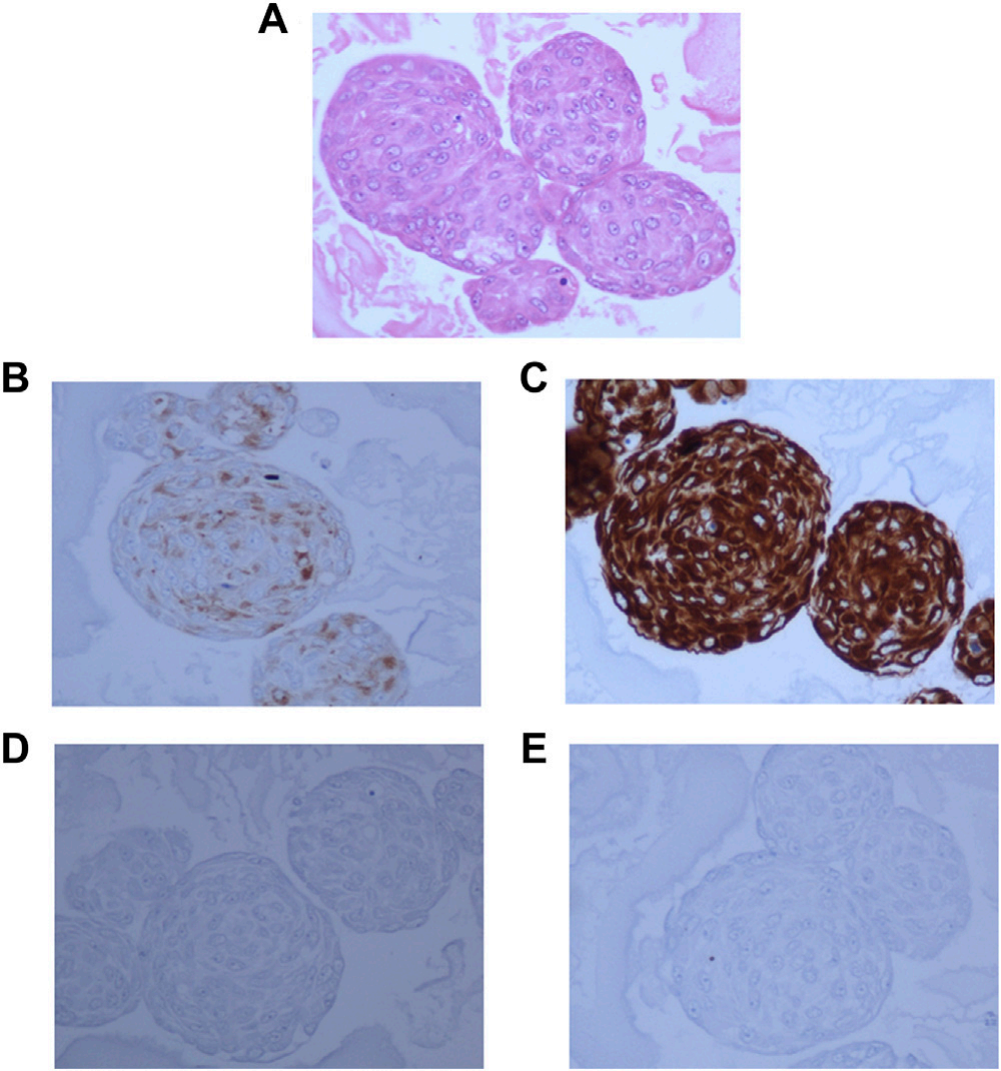
3D neurospheres sections embedded in ovalbumin and processed for FFPE blocks. **(A)**: Hematoxylin and Eosin staining showing densely packed cells throughout the neurosphere. **(B)**: Dense positive staining for Synaptophysin. **(C)**: Very strong positivity for Vimentin, **(D)**: negative staining for GFAP, and **(E)**: Very low positive staining for MIB-1 stain.

Hematoxylin and Eosin staining of the neurosphere sections showed densely packed cells (Figure 4A). The sections showed strong staining for Synaptophysin (Figure 4B), and Vimentin (Figure 4C). GFAP staining was very low in the original tumor tissue, and absent in the 3D cultures (Figure 4D) and whereas MIB-1 signal strength was low, similar to that observed in the tumor tissue (Figure 4E) indicating that the spheroids recapitulated the mixed cell lineage and marker expression seen in the tumor.

### Detection of Viable Cells in 3D Neurosphere

**FIGURE 5:**
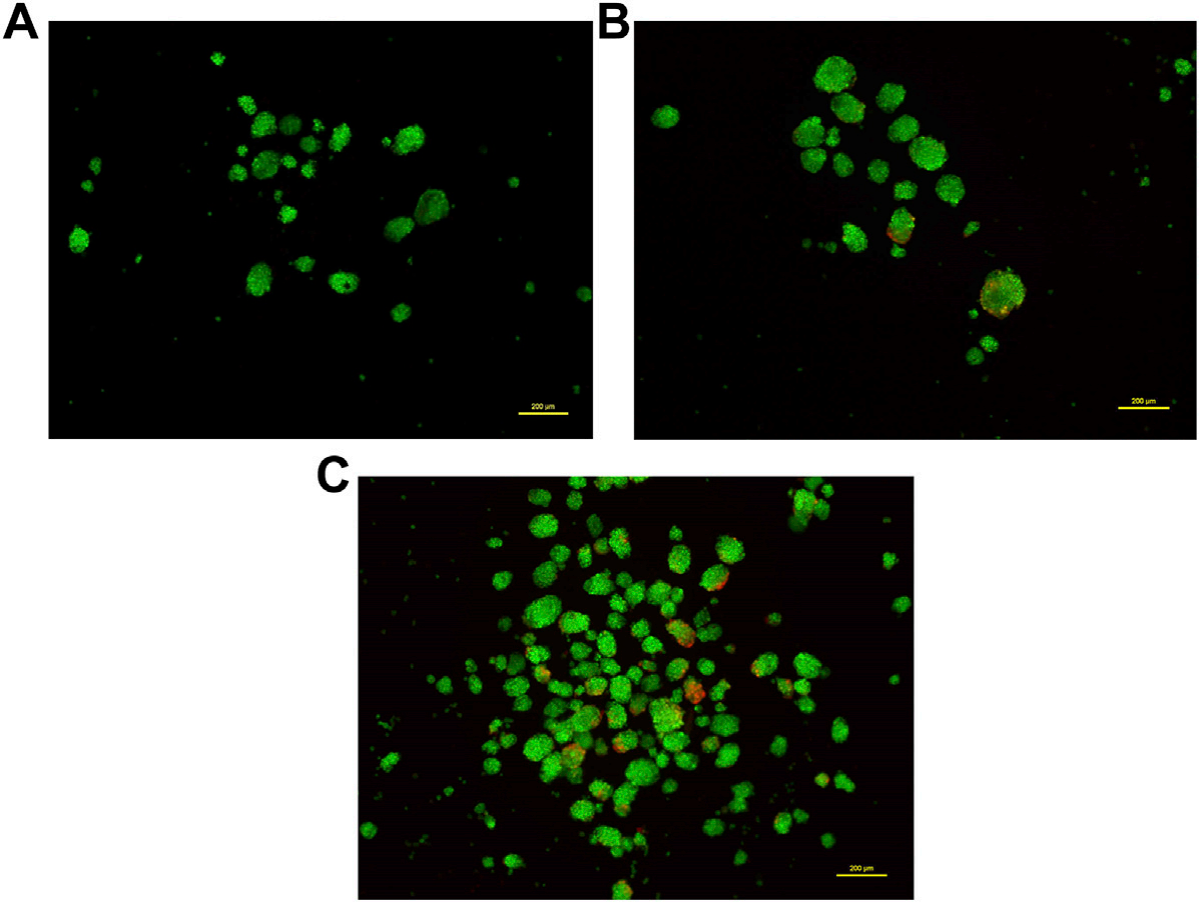
Dual staining using Calcein-AM (live cells, Green) and Propidium iodide (dead cells, Red) at different concentrations. Cells grown as spheroids at passage number 4 were used for this analysis. **(A)**: 0.5 μM Calcein-AM and 1 μM Propidium Iodide. **(B)**: 1 μM Calcein-AM and 0.5 μM Propidium Iodide. **(C)**: 1.5 μM Calcein-AM and 1 μM Propidium Iodide.

Most 3D structures have a necrotic core at the center that is surrounded by healthy and fast-growing cells. In order to determine the presence of any dead cells within the neurosphere, dual calcein-AM staining (for live cells), and propidium iodide staining (for dead cells), was employed. Spheroid cultures of cells from passage number 4 were used for this analysis. The viability of cells at different regions of the 3D spheroids was determined by capturing the images at 10 different focal planes by fluorescence microscopy. As shown in Figure 5, most of the neurosphere, including the central region, was composed of live cells with only a few dead cells. Upon quantification of the stain across multiple planes, approximately 5% cells stained for PI whereas the remaining 95% cells stained with calcein-AM confirming their viability.

### Enumeration of Cancer Stem Cells

Cancer cells cultured as 2D or monolayers typically show lower percentage of cancer stem cells (CSCs) compared to cells cultured in serum-free medium in suspension cultures that favor formation of multicellular spheroids or neurospheres. CSCs which are CD133 positive and possess self-renewal and drug resistance properties, have been successfully enriched. In order to determine the presence of cells with stem cell like properties, and their distribution in cultures grown as 2D and 3D cultures, cells from an early passage, passage 2, were evaluated for CSCs or tumor initiating cells using the CD133 marker.

**FIGURE 6:**
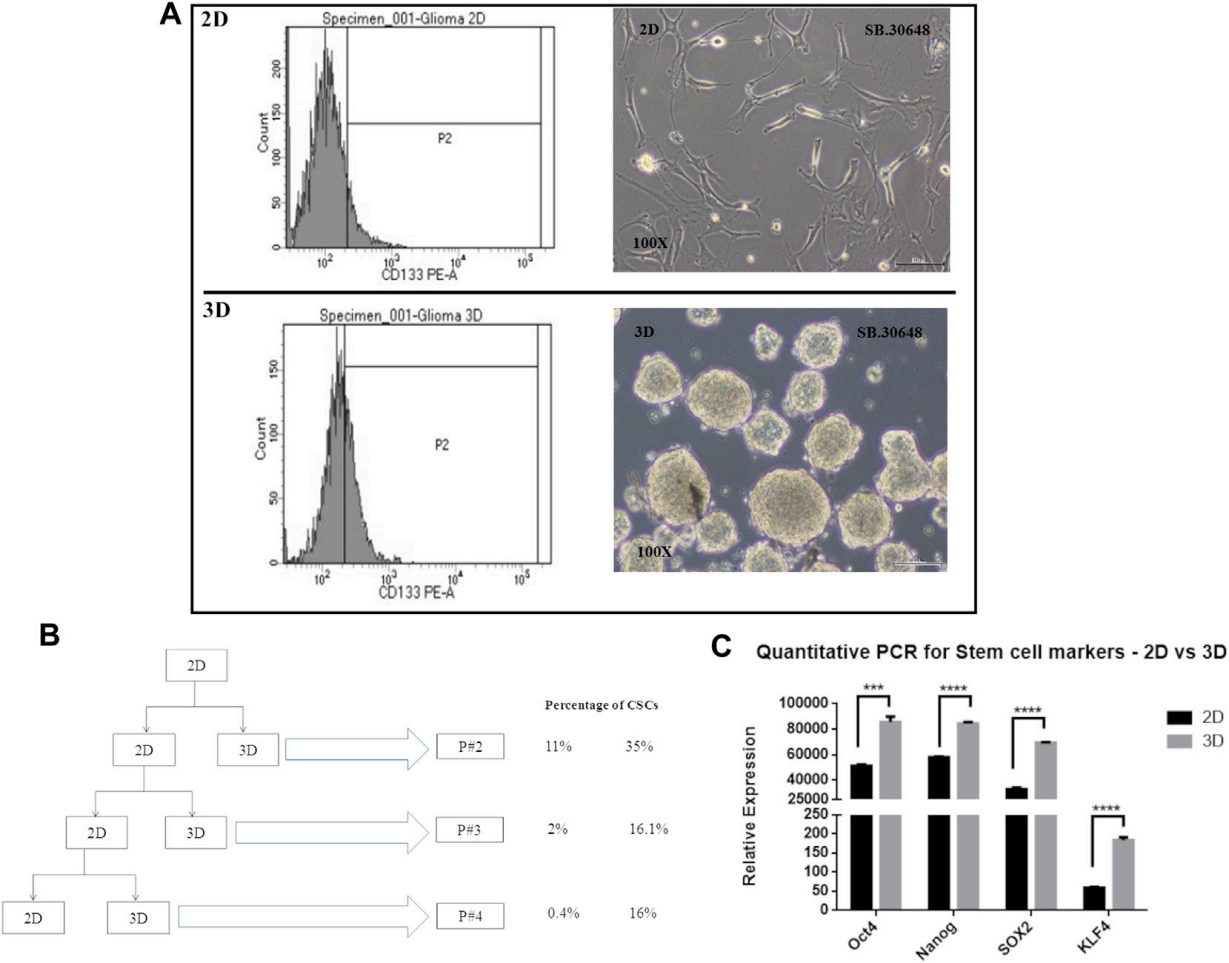
Cancer stem cell determination in 2D vs. 3D cultures. **(A)**: CD133 positive staining detected by FACS at passage 2. **(B)**: Schematic showing the passaging of the DIG cells as 2D and 3D cultures and the percentage of CD133 positive cell sin successive passages. **(C)**: Quantification of stem cell markers, Oct4, Sox2, Nanog, and KLF4 in 2D monolayer cultures vs 3D spheroid cultures by RT-PCR. Student’s *t* test (two tailed) was used to determine the statistical significance of the marker expression between the 2D and 3D cultures. *p* value less than 0.05 was considered as significant (***) represents a *p* value less than 0.005 and (****) represents a *p* value less than 0.0001.

Flow cytometric analysis was performed for CD133-stained DIG cells. The percentage of CD133-positive cells (Figure 6A) in 2D cell population was 11.8% whereas in 3D was significantly higher at 35 percent. This was repeated for successive passages until the fourth passage and showed that there were significantly higher percentages (∼16%, Figure 6B) of CSCs in 3D spheres as compared to 2D (0.4–1.8%) thereby confirming the continued generation of CSCs in 3D but not 2D cultures.

Concomitant with the identification of CSCs by CD133 expression, the expression of other commonly used stem cell markers, Sox2, Nanog, KLF, and Oct4 were quantified using RT-PCR from total RNA isolated from Passage 2 of 2D and 3D cultures. All four stem cell factors were observed to be significantly increased 1.5 to 3-fold in neurospheres compared to the monolayer cultures (Figure 6C).

### Mutation Analysis to Detect Genetic Mutations

Genes that have a role in cancer development as well as in metabolism of different chemotherapy agents were tested by NGS for gene variants (Supplementary Data). Mutations were detected in 5 genes (Table 2); of these 3, BRCA1 (S694F), MSH2 (R214I), and MSH6 (R1204Q) had nonsynonymous (missense) mutations, TSC2 (E1561*) had a stop gain mutation, and EWSR1 (P294Hfs*3) was a frameshift deletion. Of considerable importance was the observation that all three nonsynonymous mutations in BRCA1, MSH2, and MSH6 were likely to be hereditary as per COSMIC (Tate et al., 2019) and Franklin (https://franklin.genoox.com/) databases. MutS homolog 6 (MSH6) is one of the mismatch repair proteins and is encoded by the MSH6 gene, The MSH6 protein forms a heterodimer with another mismatch repair protein, MSH2. The MSH2-MSH6 heterodimeric complex is able to recognize base-base substitution and single-base insertion/deletion mismatches. Germline mutations of MSH6 are known to cause high susceptibility to glioma, as well as a number of benign or malignant tumors in other organs. Somatic MSH6 mutations result in resistance to temozolomide and may accelerate tumor progression.

**TABLE 2:** Gene mutations in DIG.

Gene	Type of mutation	Identity of mutation		Origin
		Nucleotide	Protein	
TSC2	Stop gain	G4681T	E1561*	Somatic
BRCA1	Nonsynonymous	C2081T	S694F	Hereditary
MSH2	Nonsynonymous	G641T	R214I	Hereditary
MSH6	Nonsynonymous	G3611A	R1204Q	Hereditary
EWSR1	Frameshift deletion	879delC	P294Hfs*3	Somatic

## Discussion

Phenotypic drug discovery is a very useful tool in the development of newer therapeutics for the treatment of cancers (Sharma et al., 2010). Such an approach has a major advantage in their potential to treat cancers where the mechanistic process is not completely understood or where currently approved therapies such as temozolomide are not useful for a majority of glioma patients (Moffat et al., 2017). Phenotypic drug discovery is target agnostic and can lead to the discovery of therapies that work in a particular scenario even though their mode of action may not be completely understood (Swinney and Lee, 2020). Although only surgery is usually used to treat DIG, the use of drugs in future reducing or eliminating the tumor burden in a neo-adjuvant setting can greatly benefit the patients and was one of the reasons for the establishment of the culture.

Cell culture on plastic and in serum-containing medium tends to select for certain cell types and results in a loss of the cellular complexity of the original tumor. This artefact can be minimized by culturing early passages of the patient tumor as suspension cultures in serum-free specialized medium. The cell-cell contact provides autocrine factors for growth and survival, and the presence of all the cell types of the original tumor nurtures the generation of stem cells. Cancer cells cultured as 2D or monolayers typically show lower percentage of cancer stem cells (CSCs) compared to cells cultured in serum-free medium in suspension cultures that favor formation of multicellular spheroids or neurospheres (Lee et al., 2006). Hence it is valuable to establish a panel of cell cultures from clinical brain cancer resections and use them to discover and/or validate new drug targets and drugs.

During the course of the development of such a panel of glioma cell cultures from different grades for use in screening therapeutics of interest (Supplementary Table S2), tumor tissue from a young female diagnosed with DIG was also established as a culture. The culture was a very fast-growing culture.

DIGs are rare tumors. The tumor was first described by Taratuto et al., 1982 . It was subsequently reported as “superficial cerebral astrocytoma attached to the dura” in 1984 clearly defining an entity which was previously unrecognized (Taratuto et al., 1984). Both DIA and DIG are listed together in WHO classification since both have similar features with a favorable prognosis following surgery. The terms DIA or DIG are used depending on the pattern of differentiation of the neuroepithelial component.

DIGs are generally identified in infants below the age of 2 years and very rarely in adults, with boys being more prone than girls (Per et al., 2009). However, many cases are identified in individuals after infancy perhaps due to a delay in either clinical presentation or in their identification. In a large review of 113 cases by Gelabert-Gonzalez and group (Gelabert-Gonzalez et al., 2011), 94 cases were infantile, while only 19 cases (17%), were in the non-infantile category. These tumors arise mostly in the supra-tentorial region with a predilection for the frontoparietal area (VandenBerg, 1993). Patients present with increasing head circumference and bulging fontanelle with the sunset sign (downward ocular deviation). Seizures, paresis, hyperactive reflexes may be seen (Tenreiro-Picon et al., 1995).

On CT scans, the lesions are seen as large hypodense cystic mass with solid isodense or hyperdense component (VandenBerg, 1993; Pommepuy et al., 2006). The tumors are frequently of a very large size and can measure up to 13 cm.

Histological features are characteristic, comprising of prominent reticulin-rich desmoplastic stroma within which are neoplastic astrocytes (in case of DIA), or neuronal cells (in case of DIG) (VandenBerg, 1993; Gelabert-Gonzalez et al., 2011). Aggregates of poorly differentiated cells can be seen in both lesions. The astrocytic component may include gemistiocytic forms. Mitotic activity and necrosis are uncommon, when present are noted mostly in the poorly differentiated neuroepithelial cells. On immunohistochemistry, the glial cells express both GFAP and vimentin. Synaptophysin highlights the neuronal cells. The desmoplastic stroma is positive for vimentin. MIB -1 labelling indices range from 0.5 to 5% (Tenreiro-Picon et al., 1995).

In the DIG tumor here, the tissue was predominantly made up of spindle fibroblast-like cells with scattered neoplastic astrocytes. A few of the astrocytes were gemistocytic. Focal positivity with GFAP, Synaptophysin, and Neurofilament protein confirmed the presence of mixed lineage tissue and allowed for the positive identification of DIG. There was no noticeable necrosis or mitotic foci suggesting that the tumor was well-contained and benign. There was no expression of TGFb by IHC.

Histologically, the tumors with a malignant appearance with cellular mitotically active components also have an excellent prognosis. Total surgical resection is the treatment of choice but if it cannot be achieved, chemotherapy may be effective (Duffner et al., 1994).

In our study, the entire tumor was successfully resected, and a small part of the tumor tissue was used for establishing a cell culture. Patient tissue-derived cell cultures are a potent tool in not just research into the development of novel therapy but are of direct benefit to the patients themselves as a means of personalized therapy. In addition, they are valuable tools to understand disease evolution. Rarely, DIGs have been reported to undergo transformation to malignant tumor (Al-Kharazi et al., 2013), in two cases recurring as glioblastoma multiforme (Loh et al., 2011; Prakash et al., 2014) requiring further surgery and chemotherapy. Hence establishing a cell culture was of high interest to study the likely triggers for such a transformation and to identify chemotherapy that can be of potential future use. The cells were frozen down after initial characterization. However, the cells did poorly when they were revived from a frozen stock after 3 years; since these tumors are termed “benign”, the cells may be more susceptible to death upon freeze-thawing. This has not been observed for other glioma cell cultures which we have revived even after 5 years successfully. On the other hand, a similar problem of not being able to revive a “benign” breast cancer, phyllodes, has been observed by us. One alternatively to overcome this problem with the cultures is to immortalize such rare ‘benign’ tumors with telomerase, or passaging them *in vivo* or reviving and culturing them annually, may be ways to maintain cellular viability and proliferation, and to perform chemosensitivity and other assays.

Tumors have a heterogeneous distribution and often, mutations in critical genes that drive the tumor may vary among the different parts of the tumor tissue. Though intra-tumor heterogeneity is routinely assessed in pathological diagnosis, the presence of such genetically distinct population of cells can lead to variations in the tumor response to a particular chemotherapy agent (Fisher et al., 2013). Use of tumor genome analysis can play a major role in the identification of a few driver mutations that can be implicated in the initiation of the disease and such mutations, if detected or targeted early can lead to a better prognosis in patients (Yap et al., 2012). Hence mutation analysis was done for the DIG tumor tissue which showed the presence of heritable germline mutations in the double strand break repair genes, MSH2, MSH6 and BRCA1 (Wang et al., 2000; Zhao et al., 2019). Double strand break repair is an essential component of the DNA damage repair and is needed to maintain the integrity of the genome. Mutations in the BRCA gene can lead to loss of tumor suppression function due to a lack of fidelity in DNA strand break repair during the progress of the replication fork. This can lead to further changes in the genome structure and lead to further downstream mutations leading to neoplastic growth (Zhao et al., 2019). MSH2 and 6, contribute significantly to the DNA repair mechanism during the process of replication in a complex of BRCA1 Associated Genome Surveillance Complex; BASC; (Gudmundsdottir and Ashworth, 2006). The presence of mutations in three genes, BRCA1, MSH2, and MSH6, involved in the DNA break repair can be construed as the reason for the formation of the DIG tumor in this patient. Currently there are no drugs to target these mutations but development of newer therapeutics that can modify the function of these genes holds immense value since all 3 genes have been implicated in cancers of other organs also.

BRAF mutations (V600E and V600D) reported in several cases of DIG previously (Koelsche et al., 2014; Wang et al., 2018), TP53 (R248Q) mutation which was determined to have led to a transformation of DIG to a GBM, were not identified in this case (Prakash et al., 2014). The BRAF mutation found in 4 cases of DIG in individuals that were between 10 and 14 years of age is common but not ubiquitous (Chatterjee et al., 2018). Two somatic mutations reported previously in TSC2 (E1561*), and EWSR1 (P294Hfs*3), were also found in our analysis. The significance of these mutations is unknown as a search in available databases did not reveal any instances of these mutations.

In this study, the intent was to determine the abundance of cells that have stem cell-like features in the original tumor which could pose challenges for using chemotherapy. Neurospheres were successfully established and were serially passage at least thrice. The neurospheres were characterized by IHC to determine the presence of the different type of cells that were originally present in the tumor. The entire neurosphere was coated in ovalbumin to provide a scaffold and the architecture was preserved by embedding in paraffin. The stained sections showed the expression of neuronal, glial and proliferation markers similar to that of the original tumor tissue indicating that the 3D cultures were able to capture key aspects of the original tumor. These cultures can be used as surrogates to evaluate the cellular and biochemical underpinnings of the tumor.

Given the highly proliferative nature of DIG, it was not surprising to see high percentage of CSCs even in 2D cultures compared to glioma cell lines such as U87 which in our hands yield barely 1–2% CSCs even in 3D cultures (data not shown). The percentage of CSCs was higher (16–35%) in 3D neurospheres as compared to the adherent culture, similar to the results reported for other tumors by Lee et al. and this higher number was maintained even in successive passages (Lee et al., 2006). The presence of higher number of CSCs in 3D cultures allows for the identification of drug targets and potential therapies that can modulate the self-renewal of CSCs. These cells can also be tested for their ability to form tumors in mice as a true test of CSCs and the plasticity of gliomas.

The establishment of the culture was a first step to understand why these tumors are benign with low MIB-1 positivity despite high numbers of CSCs, and why they do not metastasize or become resistant to chemotherapy drugs. Several benign tumors such as benign metastasizing meningioma, benign metastasizing leiomyoma, and a few others are known to metastasize (Patton et al., 2006) (Som et al., 1987) and understanding such mechanisms become important to eliminate the possibility of DIGs becoming malignant, especially if the entire tumor could not be resected. Of the recurred tumors, which constitute a small percentage of all DIGs, approximately 40% require additional medical, radiation, and/or further surgical intervention, and 15% of infants and children develop leptomeningeal spread or die from DIG (Hummel et al., 2012). These adverse outcomes, combined with the recognition that DIG represents a heterogeneous disease, underscore the unmet need for a deeper biological, cellular and molecular investigation using patient tissues and data to personalize the treatment and monitoring of DIG patients, as also for phenotypic screening for new drug candidates or repurposing drugs.

In conclusion, we describe a rare DIG that was successfully cultured having similar staining characteristics to the original tumor. Its mutational analysis identified previously identified hereditary gene changes such as MSH6 known to confer susceptibility to glioma, BRCA1 germline mutation which is extremely rare in gliomas, as well as a novel mutation EWSR1 in glioma. Newer drugs such as Olaparib that are approved by FDA for metastatic breast cancer patients that have inherited mutations in the BRCA1 or BRCA2 genes may be worth exploring as therapies. Also, understanding the role of BRCA1 in initiation of brain cancer is important as its role is less well understood in brain cancer. Further, Boukerroucha et al., 2015, that two breast cancer patients with hereditary BRCA1 gene mutations developed brain cancer later—hence monitoring of the DIG patient for breast and/or ovarian cancer development will be important. We believe that this is the first time that cells from DIG tumor have been successfully established in primary cultures. Such cultures and their characterization hold great promise, not only for identifying new drug targets, but also to study the recurrence or malignant transformation of rare cases of DIG.

## Data Availability

The data presented in this study are deposited in the European Variant Archive repository. The accession number for the project is PRJEB50889 and for the analyses is ERZ5173805.

## Abbreviations

CSC, Cancer stem cells; DIA, Desmoplastic infantile astrocytoma; DIG, Desmoplastic infantile ganglioglioma; FACS, Fluorescence activated cell sorting; FFPE, Formalin fixed paraffin embedded; RT-PCR, Reverse Transcriptase Polymerase Chain Reaction.

## References

[B1] Al-Kharazi K. Gillis C. Steinbok P. Dunham C. (2013). Malignant Desmoplastic Infantile Astrocytoma? A Case Report and Review of the Literature. Clin. Neuropathol. 32, 100–106. 10.5414/NP300548 23149336

[B2] Bader A. Heran M. Dunham C. Steinbok P. (2015). Radiological Features of Infantile Glioblastoma and Desmoplastic Infantile Tumors: British Columbia's Children's Hospital Experience. J. Neurosurg. Pediatr. 16, 119–125. 10.3171/2014.10.PEDS13634 25955808

[B3] Bhardwaj M. Sharma A. Pal H. K. (2006). Desmoplastic Infantile Ganglioglioma with Calcification. Neuropathology 26, 318–322. 10.1111/j.1440-1789.2006.00684.x 16961068

[B4] Boukerroucha M. Josse C. Segers K. El-Guendi S. Frères P. Jerusalem G. (2015). BRCA1 Germline Mutation and Glioblastoma Development: Report of Cases. BMC Cancer 15, 181. 10.1186/s12885-015-1205-1 25880076 PMC4377178

[B5] Chandrashekhar T. N. Mahadevan A. Vani S. Yasha T. C. Sampath S. Chandramouli B. A. (2012). Pathological Spectrum of Neuronal/glioneuronal Tumors from a Tertiary Referral Neurological Institute. Neuropathology 32, 1–12. 10.1111/j.1440-1789.2011.01206.x 21410777

[B6] Chatterjee D. Garg C. Singla N. Radotra B. D. (2018). Desmoplastic Non-infantile Astrocytoma/ganglioglioma: Rare Low-Grade Tumor with Frequent BRAF V600E Mutation. Hum. Pathol. 80, 186–191. 10.1016/j.humpath.2018.06.005 29902580

[B7] Craver R. D. Nadell J. Nelson J. S. (1999). Desmoplastic Infantile Ganglioglioma. Pediatr. Dev. Pathol. 2, 582–587. 10.1007/s100249900166 10508884

[B8] Derinkuyu B. E. Ucar M. Borcek A. O. Damar C. Oztunali C. Gul Alimli A. (2015). Non-infantile Variant of Desmoplastic Ganglioglioma: Conventional and Advanced MR Imaging Characteristics. Neuroradiol. J. 28, 259–263. 10.1177/1971400915595579 26246092 PMC4757299

[B9] Duffner P. K. Burger P. C. Cohen M. E. Sanford R. A. Krischer J. P. Elterman R. (1994). Desmoplastic Infantile Gangliogliomas: an Approach to Therapy. Neurosurgery 34, 583–589. 10.1227/00006123-199404000-00003 8008154

[B10] Fisher R. Pusztai L. Swanton C. (2013). Cancer Heterogeneity: Implications for Targeted Therapeutics. Br. J. Cancer 108, 479–485. 10.1038/bjc.2012.581 23299535 PMC3593543

[B11] Gelabert-Gonzalez M. Serramito-García R. Arcos-Algaba A. (2011). Desmoplastic Infantile and Non-infantile Ganglioglioma. Review of the Literature. Neurosurg. Rev. 34, 151–158. 10.1007/s10143-010-0303-4 21246390

[B12] Gessi M. Pietsch T. (2013). The Diagnostic Role and Clinical Relevance of Determination of BRAF Status in Brain Tumors. Per. Med. 10, 405–412. 10.2217/pme.13.27 29783415

[B13] Gudmundsdottir K. Ashworth A. (2006). The Roles of BRCA1 and BRCA2 and Associated Proteins in the Maintenance of Genomic Stability. Oncogene 25, 5864–5874. 10.1038/sj.onc.1209874 16998501

[B14] Hummel T. R. Miles L. Mangano F. T. Jones B. V. Geller J. I. (2012). Clinical Heterogeneity of Desmoplastic Infantile Ganglioglioma: a Case Series and Literature Review. J. Pediatr. Hematol. Oncol. 34, e232–6. 10.1097/MPH.0b013e3182580330 22735886

[B15] Khaddage A. Chambonniere M. L. Morrison A. L. Allard D. Dumollard J. M. Pasquier B. (2004). Desmoplastic Infantile Ganglioglioma: A Rare Tumor with an Unusual Presentation. Ann. Diagn. Pathol. 8, 280–283. 10.1016/j.anndiagpath.2004.07.004 15494934

[B16] Koelsche C. Sahm F. Paulus W. Mittelbronn M. Giangaspero F. Antonelli M. (2014). BRAF V600E Expression and Distribution in Desmoplastic Infantile Astrocytoma/ganglioglioma. Neuropathol. Appl. Neurobiol. 40, 337–344. 10.1111/nan.12072 23822828

[B17] Lee J. Kotliarova S. Kotliarov Y. Li A. Su Q. Donin N. M. (2006). Tumor Stem Cells Derived from Glioblastomas Cultured in bFGF and EGF More Closely Mirror the Phenotype and Genotype of Primary Tumors Than Do Serum-Cultured Cell Lines. Cancer Cell 9, 391–403. 10.1016/j.ccr.2006.03.030 16697959

[B18] Li Z. (2013). CD133: a Stem Cell Biomarker and beyond. Exp. Hematol. Oncol. 2, 17. 10.1186/2162-3619-2-17 23815814 PMC3701589

[B19] Loh J. K. Lieu A. S. Chai C. Y. Howng S. L. (2011). Malignant Transformation of a Desmoplastic Infantile Ganglioglioma. Pediatr. Neurol. 45, 135–137. 10.1016/j.pediatrneurol.2011.04.001 21763958

[B20] Moffat J. G. Vincent F. Lee J. A. Eder J. Prunotto M. (2017). Opportunities and Challenges in Phenotypic Drug Discovery: an Industry Perspective. Nat. Rev. Drug Discov. 16, 531–543. 10.1038/nrd.2017.111 28685762

[B21] Patton K. T. Cheng L. Papavero V. Blum M. G. Yeldandi A. V. Adley B. P. (2006). Benign Metastasizing Leiomyoma: Clonality, Telomere Length and Clinicopathologic Analysis. Mod. Pathol. 19, 130–140. 10.1038/modpathol.3800504 16357844

[B22] Paulus W. Schlote W. Perentes E. Jacobi G. Warmuth-Metz M. Roggendorf W. (1992). Desmoplastic Supratentorial Neuroepithelial Tumours of Infancy. Histopathology 21, 43–49. 10.1111/j.1365-2559.1992.tb00341.x 1634201

[B23] Per H. Kontaş O. Kumandaş S. Kurtsoy A. (2009). A Report of a Desmoplastic Non-infantile Ganglioglioma in a 6-Year-Old Boy with Review of the Literature. Neurosurg. Rev. 32, 369–374. 10.1007/s10143-009-0195-3 19280238

[B24] Pommepuy I. Delage-Corre M. Moreau J. J. Labrousse F. (2006). A Report of a Desmoplastic Ganglioglioma in a 12-Year-Old Girl with Review of the Literature. J. Neurooncol. 76, 271–275. 10.1007/s11060-005-6500-2 16205962

[B25] Prakash V. Batanian J. R. Guzman M. A. Duncavage E. J. Geller T. J. (2014). Malignant Transformation of a Desmoplastic Infantile Ganglioglioma in an Infant Carrier of a Nonsynonymous TP53 Mutation. Pediatr. Neurol. 51, 138–143. 10.1016/j.pediatrneurol.2014.02.012 24768217

[B26] Sharma S. V. Haber D. A. Settleman J. (2010). Cell Line-Based Platforms to Evaluate the Therapeutic Efficacy of Candidate Anticancer Agents. Nat. Rev. Cancer 10, 241–253. 10.1038/nrc2820 20300105

[B27] Smith S. H. (2006). “Uncommon Pediatric Brain Tumours,” in, eds. Rhagavan D. Brecher M. L. Johnson D. H. Meropol N. J. Moots P. L. Rose P. G. (John Wiley & Sons), 878. Available at: https://www.wiley.com/en-us/Textbook+of+Uncommon+Cancer%2C+3rd+Edition-p-9780470030554 .

[B28] Som P. M. Sacher M. Strenger S. W. Biller H. F. Malis L. I. (1987). “Benign” Metastasizing Meningiomas. AJNR. Am. J. Neuroradiol. 8, 127–130. Available at: http://www.ncbi.nlm.nih.gov/pubmed/3101455 . 3101455 PMC8334031

[B29] Sutherland R. M. (1988). Cell and Environment Interactions in Tumor Microregions: the Multicell Spheroid Model. Science 240, 177–184. 10.1126/science.2451290 2451290

[B30] Swinney D. C. Lee J. A. (2020). Recent Advances in Phenotypic Drug Discovery. F1000Res 9, 944. 10.12688/f1000research.25813.1 PMC7431967 32850117

[B31] Taratuto A. L. Monges J. Lylyk P. Leiguarda R. (1984). Superficial Cerebral Astrocytoma Attached to Dura. Report of Six Cases in Infants. Cancer 54, 2505–2512. 10.1002/1097-0142(19841201)54:11<2505:aid-cncr2820541132>3.0.co;2-g 6498740

[B32] Taratuto A. L. Monges J. Lylyk P. Leiguarda R. (1982). “Meningocerebral Astrocytoma Attached to Dura with Desmoplastic Reaction,” in Proceedings of the IX congress of Neuropathology (Vienna, 5–10.

[B33] Tate J. G. Bamford S. Jubb H. C. Sondka Z. Beare D. M. Bindal N. (2019). COSMIC: the Catalogue of Somatic Mutations in Cancer. Nucleic Acids Res. 47, D941–D947. 10.1093/nar/gky1015 30371878 PMC6323903

[B34] Tenreiro-Picon O. R. Kamath S. V. Knorr J. R. Ragland R. L. Smith T. W. Lau K. Y. (1995). Desmoplastic Infantile Ganglioglioma: CT and MRI Features. Pediatr. Radiol. 25, 540–543. 10.1007/BF02015789 8545186

[B35] VandenBerg S. R. (1993). Desmoplastic Infantile Ganglioglioma and Desmoplastic Cerebral Astrocytoma of Infancy. Brain Pathol. 3, 275–281. 10.1111/j.1750-3639.1993.tb00754.x 8293187

[B36] Wang A. C. Jones D. T. W. Abecassis I. J. Cole B. L. Leary S. E. S. Lockwood C. M. (2018). Desmoplastic Infantile Ganglioglioma/Astrocytoma (DIG/DIA) Are Distinct Entities with Frequent BRAFV600 Mutations. Mol. Cancer Res. 16, 1491–1498. 10.1158/1541-7786.MCR-17-0507 30006355 PMC7269191

[B37] Wang Y. Cortez D. Yazdi P. Neff N. Elledge S. J. Qin J. (2000). BASC, a Super Complex of BRCA1-Associated Proteins Involved in the Recognition and Repair of Aberrant DNA Structures. Genes Dev. 14, 927–939. Available at: http://www.ncbi.nlm.nih.gov/pubmed/10783165 . 10.1101/gad.14.8.927 10783165 PMC316544

[B38] Yap T. A. Gerlinger M. Futreal P. A. Pusztai L. Swanton C. (2012). Intratumor Heterogeneity: Seeing the Wood for the Trees. Sci. Transl. Med. 4, 127ps10. 10.1126/scitranslmed.3003854 22461637

[B39] Zhao W. Wiese C. Kwon Y. Hromas R. Sung P. (2019). The BRCA Tumor Suppressor Network in Chromosome Damage Repair by Homologous Recombination. Annu. Rev. Biochem. 88, 221–245. 10.1146/annurev-biochem-013118-111058 30917004 PMC7004434

