# Supplementary material for: Establishment and Characterization of Brain Cancer Primary Cell Cultures From Patients to Enable Phenotypic Screening for New Drugs
Source: Front Pharmacol. 2022 Mar 17;13:778193. doi: 10.3389/fphar.2022.778193 (PMC8970592; doi:10.3389/fphar.2022.778193)
Supplement: Supplementary file 2 [file Table1.docx]

Supplementary data:

List of genes targeted in a custom panel for mutation analysis

| ABCB1 | AR | CYP1A2 | CYP3A5 | ERCC4 | HRAS | MSH2 | PDGFRA | TOP1 |
| --- | --- | --- | --- | --- | --- | --- | --- | --- |
| ABCC1 | BCR | CYP1B1 | DCK | ERCC5 | JAK1 | MSH6 | PDGFRB | TP53 |
| ABCC2 | BRAF | CYP24A1 | DDB1 | ESR1 | JAK2 | MTHFD1 | PGR | TSC1 |
| ABCC3 | BRCA1 | CYP27B1 | DYNC2H1 | EWSR1 | JAK3 | MTHFR | PIK3CA | TSC2 |
| ABCC4 | BRCA2 | CYP2B6 | EGFR | EZH2 | KDR | MTOR | PTEN | VHL |
| ABCC5 | CCND1 | CYP2C19 | EML4 | F2R | KIT | NF1 | REL | XRCC1 |
| ABCG2 | CCND2 | CYP2C8*3 | ERRB2 | FGFR4 | KRAS | NR1I2 | RET |  |
| ABL1 | CDA | CYP2C9 | ERCC1 | FLT3 | LINS1 | NR1I3 | ROS1 |  |
| AKT1 | CYP19A1 | CYP2E1 | ERCC2 | GSTA1 | MET | NRAS | RRM1 |  |
| ALK | CYP1A1 | CYP3A4 | ERCC3 | GSTP1 | MLH1 | PARP1 | TERT |  |
